# Recent Advances, Opportunities, and Challenges in Developing Nucleic Acid Integrated Wearable Biosensors for Expanding the Capabilities of Wearable Technologies in Health Monitoring

**DOI:** 10.3390/bios12110986

**Published:** 2022-11-08

**Authors:** Mohammad Janghorban, Irvyne Aradanas, Sara Kazemi, Philippa Ngaju, Richa Pandey

**Affiliations:** 1 Department of Biomedical Engineering, University of Calgary, Calgary, AB T2N 1N4, Canada; 2 Hotchkiss Brain Institute, University of Calgary, Calgary, AB T2N 1N4, Canada

**Keywords:** wearable biosensors, nucleic acid assays, aptamers, CRISPR-Cas, fabrication

## Abstract

Wearable biosensors are becoming increasingly popular due to the rise in demand for non-invasive, real-time monitoring of health and personalized medicine. Traditionally, wearable biosensors have explored protein-based enzymatic and affinity-based detection strategies. However, in the past decade, with the success of nucleic acid-based point-of-care diagnostics, a paradigm shift has been observed in integrating nucleic acid-based assays into wearable sensors, offering better stability, enhanced analytical performance, and better clinical applicability. This narrative review builds upon the current state and advances in utilizing nucleic acid-based assays, including oligonucleotides, nucleic acid, aptamers, and CRISPR-Cas, in wearable biosensing. The review also discusses the three fundamental blocks, i.e., fabrication requirements, biomolecule integration, and transduction mechanism, for creating nucleic acid integrated wearable biosensors.

## 1. Introduction

Wearable sensors are “on-body” devices that are designed to collect essential data on human health (electrical, electrophysiological, biochemical) [1]. Their popularity is reflected by a rise in their usage in the last few years, driven by consumers’ increasing desire to monitor their health and vital signs [2]. These wearable devices have the potential to enable preventive, time-sensitive, and patient-centered decision-making, especially in remote communities, by providing useful health data on early onset and progression of diseases related to infection, mental health and neurological disorders, rare and chronic disease, etc. [3,4,5,6,7]. Wearable sensing has numerous on-field applications, such as tracking the health of military personnel in hard-to-reach areas, astronauts on space missions, congregated populations in remote areas, healthcare workers, and the general population during endemic or pandemic circumstances [7]. Utilization of sophisticated medical technology for a comprehensive biochemical profile of patient samples necessitates trained staff, complex operation, expensive equipment, and access to reliable electricity [8]. Many of these challenges are alleviated by point-of-care (POC) diagnostic devices that facilitate rapid and facile operation at the site of need [9]. However, many applications, such as screening for the outbreak, monitoring during surgery, and detection of biohazards, need continuous and aseptic monitoring to avoid the possibility of infection spread or sample contamination [10]. In these scenarios, wearable biosensors have the potential to be quickly modified for a specific target, such as viruses or bacteria, and readily be transformed into “easy to wear” technology [11]. Currently, most commercially viable wearable devices can assess physical indications (e.g., temperature, activity, tension, etc.) and electrophysiological activities (e.g., electroencephalography, electrocardiography, electromyography, etc.) [12]. However, they do not provide more insightful information on users’ health status at the bio-molecular level, such as nutrients, hormones, proteins, small molecules, enzymes, etc. [13]. As wearable technology becomes increasingly mainstream [14], it necessitates the development of targeted applications to create wearable devices with molecular diagnostics capability. These biosensors can detect molecular analytes from various biological fluids, such as sweat, interstitial fluid, saliva, and tears, using various sensing mechanisms [14]. To function as a wearable biosensor, the device needs biorecognition components that are sensitive, specific, stable, consistent, and easy to functionalize. State-of-the-art wearable biosensors are limited to protein-based (enzymes and antibody) [15] chemical sensing [16] or specifically targeting diabetes (glucose) management [17]. The wearable applications of these sensors require their long-term continuous usage in fluctuating temperatures, pH, and the environment. For example, enzyme-based wearable biosensors suffer from limited stability due to their reaction byproducts and fluctuation due to temperature, hence making it challenging to obtain reliable signal generation continuously [18]. Nucleic acids (NAs) have recently gained popularity as biological recognition elements in wearable biosensors. NA-based biosensors are capable of binding with a target biomarker (affinity) and hybridizing with them (strand hybridization, displacement) [19]. In comparison to protein-based biorecognition elements, NAs are more scalable due to their chemical synthesis not needing any host animals [20], can overcome the multi-step processing requirements due to their programmability, have compatibility with a wide range of conjugation chemistries, and have the structure switching ability that can process reversible signal generation for continuous monitoring [21]. Some NAs, specifically developed to function as an enzyme (DNAzyme, ribozymes) or antibody (Aptamer), are synthesized using the biochemical in-vitro selection process for identifying a specific specimen in the biological sample. This process does not require prior knowledge of the molecular target binding with the sequences. This combines biomarker discovery and probe development into a single step and expedites molecular diagnostic assay development [22]. To alleviate some of these challenges in protein-based biosensing approaches, there has been a growing interest in combining NA and specifically functional NAs with POC devices [23], which has encouraged wearable biosensor researchers to explore unique NA-integrated assays for detecting non-invasive health-related biomarkers [24]. NA-based biosensors offer excellent analytical performance when used with different transduction mechanisms to develop reagent-less and wash-free assays [25]. However, challenges remain in understanding their usage in continuous and wearable applications [26].

Herein, we provide a narrative review of the most recent advances, especially in the last decade, in nucleic acid-based wearable biosensing and their prospects for wearable device development and medical application. We review three key areas of nucleic acid integrated wearable device development: (1) fabrication requirements, (2) bio recognition specifically focusing on nucleic acids, and (3) sensing mechanism. We take one of the main goals in wearable biosensing, namely, to produce integrated POC technologies, as a reference to discuss progress, challenges, and opportunities in the three areas.

## 2. Fabrication Requirements

**Figure 1:**
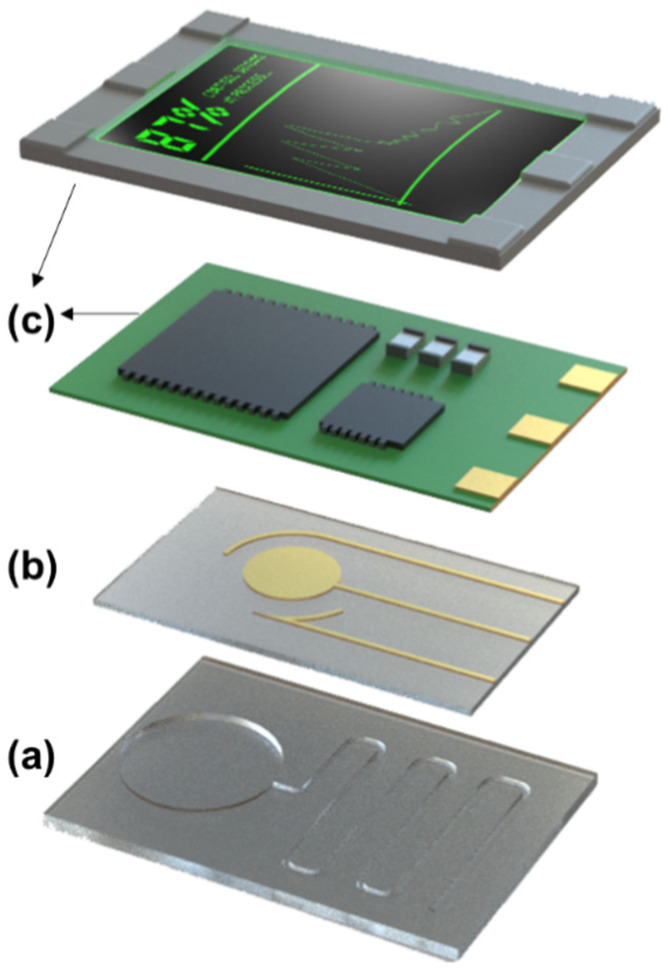
General schematic of different components of a wearable biosensor: (**a**) microfluidic/reagent layer, (**b**) sensing layer, (**c**) packaging layer.

Generally, the fabrication of a nucleic acid integrated wearable biosensor involves the assembly of three core layers crucial for the meticulous operation of the wearable device [27]. These three layers are commonly the microfluidic/reagent layer, the sensing layer, and the readout/packaging layer (Figure 1).

Depending on the biosensor’s function, other layers may be added, or existing layers might be altered to incorporate new components. For example, recombinase polymerase amplification (RPA) reagents are widely used for biomarkers or sensing probe amplification in a diagnostic assay and can be incorporated into the microfluidic channel to further enhance the detection limit. Generally, the first layer is usually a flexible polymer-based material, while the second layer includes metal or semi-conducting materials, and eventually, the third layer entails the printed circuit board (PCB) and packaging components [28]. A variety of fabrication techniques ranging from lithography to additive manufacturing, can be used for the fabrication of each layer.

### 2.1. Microfluidics/Reagent Layer

**Figure 2:**
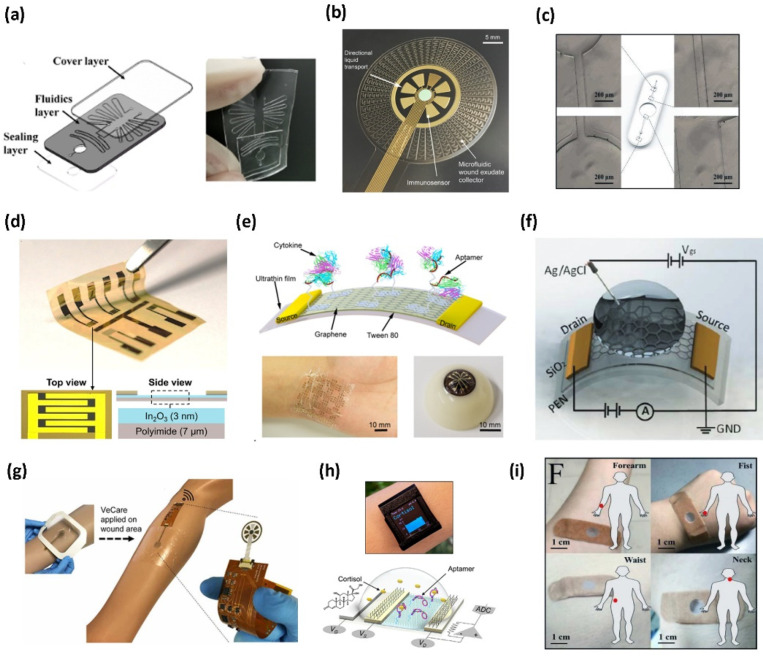
Different approaches of creating the three-layer structure of a wearable biosensor. The microfluidic layer can be embedded in a broad range of polymers. (**a**) Microfluidics was patterned and assembled using three layers of PDMS (Reprinted with permission from Ref. [31] Copyright 2021, John Wiley and Sons). (**b**) Polyurethane microfluidic pattern produced by photolithography (Reprinted with permission from Ref. [29] Copyright 2021, the authors). (**c**) Ecoflex microfluidic produced by micromachined mold (Reprinted with permission from Ref. [30] Copyright 2019, Elsevier). The sensing layer with the sensing elements, such as the electrodes, is also assembled on flexible polymers. The bilayer structure of the sensing layer is achieved by a combination of deposition techniques. (**d**) Sol-gel chemistry to produce a thin layer of Indium oxide (reprinted with permission from Ref. [27] Copyright 2021, the authors), (**e**) E-beam deposited chromium and gold source and drain electrodes (reprinted with permission from Ref. [41] Copyright 2020, the authors), and (**f**) titanium and gold-sputtered drain and source electrodes (reprinted with permission from Ref. [42] Copyright 2021, Royal Society of Chemistry). The third layer is the readout packaging layer of the product. This layer can be (**g**) a perforated wound dressing (reprinted with permission from Ref. [29] Copyright 2021, the authors), (**h**) integrated into a wristwatch (reprinted with permission from Ref. [27] Copyright 2021, the authors), or (**i**) attached to the skin via a bandage-like structure (reprinted with permission from Ref. [30] Copyright 2019, Elsevier).

The microfluidic layer is responsible for delivering fluidics to the sensing array, holding and mixing reagents. Depending on their application and transduction mechanism (optical, electrical, or electrochemical), nucleic-acid based assays can be either integrated into the microfluidic channels to be mixed with biofluids or transfer reagents containing analytes to the sensing layer where the nucleic acids arrays are immobilized [29,30]. The microfluidics or the reagent integration layer (Figure 1a) is often made of a polymer, such as polyethylene terephthalate (PET) or polybutylene adipate terephthalate (PBAT or Ecoflex), and is inherently flexible. The polymer needs to be in constant contact with the user’s skin to facilitate the collection of the necessary biomarkers for sensing or to store other reagents needed to amplify the detection abilities [29]. Figure 2a–c presents some recent studies on producing microfluidic layers using silicone-based materials [29,30]. The typical fabrication of the microfluidics layer involves designing a master mold using soft lithography techniques. The process usually requires spin coating a rigid substrate with a photoresist. Then, a mask is applied to the coated substrate and inserted into a mask aligner machine to make negative or positive patterns of microfluidic design. Using this process, a master mold for a three-layer microfluidic chamber was fabricated for nucleic acid detection [31]. The first and the third layer would encapsulate the microfluidics on the second layer and seal the channels. The second layer with the microfluidic channels is produced using the master mold. This master mold was used with a mixture of Polydimethylsiloxane (PDMS) precursors and curing agents to develop the fluidics prototype. Another thin layer was fabricated to cover the microfluidic channel for reagent integration using an oxygen plasma treatment (Figure 2a) [31]. The silanol groups (-OH) on the surface of the PDMS layers are exposed more after plasma treatment, and once they are brought together with other PDMS layers, they create strong covalent (Si-O-Si) connections. A virtually unbreakable seal between the layers is created by these covalent connections. Nevertheless, to reduce the total cost of the manufacturing process, the plasma treatment step can also be skipped by using the adjusted ratio of PDMS pre-polymer and curing agent mixture to act as glue between layers. In this manner, the microfluidic channel is reversibly attached to the covering layer by only the increased friction force and soft contact of the PDMS, which would result in the repetitive usage of the microfluidic channel with new reagents; however, this will require aggressive cleaning of it for efficient biochemistry [32]. Photolithography also facilitates the design and development of complicated patterns of microfluidics that are inspired by nature, such as the wound exudate collector, without needing to produce a mold to assemble the microfluidics and integrate them with sensing electrodes to miniaturize the total dimension of the device (Figure 2b). A thin layer of Ni with a thickness of 25 nm is first sputtered on a Si Wafer. This layer is then spin-coated with SU-8 photoresist to be patterned using photolithography. Gold and silver thin films are then deposited on these layers using thermal and e-beam evaporators. Following the silver deposition, 0.1 M of FeCl_3_ is drop cast on the silver thin film to produce the Ag/AgCl reference electrode. By spin coating, another layer of SU-8 and using photolithography, the wound exudate collector is then patterned, and the whole stack is released from the Si wafer by etching the Ni layer with FeCl_3_ [29]. Apart from soft lithography, other microfabrication methods, such as mechanical micromachining, can be used to make a silicon master mold. Silicon can be mechanically micromachined using end mills or profiled cutting tools via xurography. For diamond tools on end mills, the minimum diameter of the end mills is 200 µm, and for tungsten carbide tools, it is approximately 50 µm, rendering fluidics sizes of tens of micrometers. Xurography is a method for directly producing microfluidic channels, masks, or molds from thin film polymer materials without the need for cleanroom conditions [33]. Ecoflex-based fluidics devices can also be fabricated from these molds, and the cured pieces can later be assembled using plasma treatment (Figure 2c) [30]. However, depending to the shape of the mold, more simple production procedures can be used. In a recent study, a double-layer polymethyl methacrylate (PMMA) mold was developed by mounting the fluidic patterns on a supporting base with a double-sided adhesive (DSA) and utilizing it as a cast for another PDMS-based component [34]. Conventionally, polymer-based microfluidics layers rely on clean room-based fabrication techniques and/or predominant use of non-biodegradable polymer supporting substrates, as already described (i.e., polyimide [PI], PET, and silicone elastomers). The challenge with these approaches is a limited commercial translation due to the high manufacturing cost per unit. Next-generation non-invasive wearables are developing into skin-friendly (e.g., breathable), customizable, re-attachable, and designed for one-time use to minimize the risks of inflammation and infections [35,36]. Paper-based wearable electronics are excellent candidates for this as they are intrinsically sustainable, breathable, flexible, biocompatible, and biodegradable, hence showing potential for promising and versatile wearable applications [37]. For fluidics applications, paper-based devices are treated with chemical molecules that can create fluidic patterns by making the surface affinity amenable to fluid. Sadri et al. demonstrated the use of fluoroalkylated trichlorosilane to the surface chemistry of cellulose fibers, rendering paper omniphobic (resistant to wetting to aqueous solutions and organic liquids) while preserving mechanical flexibility, strength, and breathability of untreated paper. Omniphobic paper has been used to fabricate various low-cost microfluidic devices for PoC diagnostics, among other applications, and can be adapted in wearable biosensors [38].

### 2.2. Sensing Layer

Sensing elements are the transducers mounted on top of the microfluidics/reagent layer to receive the analyte biomarkers for biorecognition. The sensing layer is commonly integrated with the rest of the electrical circuit to transmit the signals to the reader. However, the sensing components can also be integrated into the microfluidic layer to reduce the general dimensions of the final device or enhance the analytical performance of the sensor [27,29]. Mostly, the electrodes are conductive films patterned using thin film deposition techniques mounted on a flexible layer, such as Mylar thin film, polyamide, and SiO_2_ coated polyethylene naphthalate (PEN), creating a bilayer that can be attached to a microfluidic layer (Figure 2d–f). Depending on the sensing layer material (metal, carbon nanomaterial, polymer) and the transduction mechanism, patterned electrodes on the sensing layer can be functionalized with nucleic acids using various immobilization strategies [39,40,41,42,43,44,45]. For instance, NA attachment to gold electrodes happens most commonly through the chemical bonding between the gold and thiol modified 5′ or 3′ end of the NA [39,40]. However, this strategy might not be applicable to the colorimetric detection of some targets. For instance, the general process for producing wearable Graphene-based transistors is commonly initiated by transferring a thin Graphene layer to the polymer substrate. This thin layer is typically grown on a thin metal film such as copper or nickel. To release the Graphene on a specific substrate, the thin metal film should be etched, while at the same time, the Graphene layer remains attached to the substrate’s surface. Quite often, a carrier film is deposited by spin-coating a thin layer of sacrificial polymer (PMMA) on the top of the metal sheet and is removed without damaging the monolayer [41,46,47]. For most graphene-based electrodes, the NA functionalization occurs through a crosslinker such as 1-pyrenebutanoic acid succinimidyl ester, requiring a crosslinking agent to immobilize probes on the electrode [41,42].

Subsequently, a combination of lithography and other deposition techniques, such as E-beam evaporation, sputtering, or inkjet printing, is also utilized to pattern the sensing electrodes onto the polymeric substrates for creating field effect transistor (FET)-based wearable devices. For example, Wang et al. developed an FET-based wearable biosensor using a thin layer of indium oxide (In_2_O_3_) precursors coated on a polyimide substrate using sol-gel chemistry (Figure 2d) [27]. Due to their quick response times for real-time and continuous monitoring, large detectable concentration range, high sensitivity, high consistency for reliable sensing, and ability to integrate with other microfluidic, nanobiosensors based on In_2_O_3_, FETs are well suited for wearable biosensor applications. In_2_O_3_ thin film also has a 2D structure that increases the surface-to-volume ratio for the semi-conducting channel. Furthermore, the In_2_O_3_ nanobiosensors are excellent for multiplexed sensing because of the ease with which the exposed semiconductor channel areas can be modified with different functional groups or receptors [27,48]. In another study, chromium and gold were utilized to pattern drain, source, and gate electrodes on a thin layer of Mylar film using a combination of lithography and E-beam evaporation. Eventually, a thin graphene layer was transferred onto the substrate by a PMMA carrier layer (Figure 2e) [41]. The PMMA then could easily be dissolved by acetone, leaving the monolayer graphene sheet behind [41]. Similarly, a 50 nm layer of SiO_2_ was sputtered on a polyethylene naphthalate (PEN) substrate that would act as the base for the photolithography process. The source and drain electrodes were produced afterward using E-beam evaporation of titanium and gold, and a monolayer of Graphene was transferred onto the organic PEN layer as the sensing electrode (Figure 2f) [42]. One of the important aspects of the sensing layer is the consideration for the biorecognition element immobilization. These layers need to be compatible with the functionalization chemistry without affecting its transduction performance [49] many different sensing materials have been utilized, such as gold thin films [29,41,50,51] and graphene monolayers. Materials such as carbon nanomaterials, although cost-effective and easy to fabricate, pose limitations such as electrical performance deterioration and high electrical resistance. They also need another functionalization layer deposition to form bonds with biomolecules. On the other hand, metal nanomaterials and thin films such as gold thin layers have cost limitations, albeit their high compatibility and biomolecule functionalization through thiol bonding for NA conjugation and biosensing. The future entails fabricating the sensing layers as part of the flexible polymer layer and the microfluidics layer—a method frequently adopted in the discrete flexible point-of-care technology [52] or wearable strain, force, and pressure sensors [53,54].

### 2.3. Readout/Packaging Layer

The electronic readout layer has a packaging and electronic interfacing role for the wearable device. It insulates the sensor from unintentional noise, acts as a protective cover [27], and provides the necessary front-end analog electronic circuit for readout purposes. Depending on their application, there are various materials, such as printed circuit boards (PCB) and integrated circuits (IC) [27,29], needed for their fabrication. This layer can be in the form of a patch that encapsulates the rest of the product and facilitates the attachments of the device and the sensing elements to the user’s skins (Figure 2g) [29]. Aside from implementing the sensing electrodes in a microfluidic wound exudate collector, Gao et al. also packed the sensing components into a breathable barrier and a wound contact layer to set up the wound dressing for biomolecule analysis on a patient with a venous ulcer [29]. It can also be produced in the shape of a wristwatch with liquid-crystal display (LCD) and other electronic components (Figure 2h) [27]. The use of wireless modules in flexible printed circuit boards (FPCB) for wireless transmission of data produced by the sensing array. In this study, the disposable sensing array was connected to the FPCB, and an LCD monitor was implemented into the circuit board to show the generated signals from the sensor array. This wearable biosensor, including the rest of the electronic components, is designed to be integrated into a smartwatch so it can be easily carried by the patient (Figure 2h) [27]. The electronic components involve a potentiostat chip that helps to analyze the data received from the sensor, a digital to analog converter, Bluetooth module, and a microcontroller unit that does the main processing of the obtained data [27]. To improve the biosensor’s sustainability over an extended period of time, there is a need to design self-powering biosensors and implement energy harvesting mechanisms into their wearable biosensing devices [55]. There have been efforts to develop triboelectric or thermo-electric power generators that utilize bodily functions to generate power for wearable devices [56,57]. However, output signals with higher noise, as well as low power output, might inhibit the usage of some transduction and readout mechanisms, such as electrochemical or electrical devices. For example, some of the produced noises in common three-electrode chronoamperometry devices stem from the alternations in generated voltage or the potential difference between the working and reference electrodes, limiting their practicality in analytical performance [58]. Nevertheless, other electrochemical approaches also need the use of a potentiostat, which necessitates the use of a stable power source, forcing researchers to make further improvements to their device design.

While these three layers are very popular in electrical and electrochemical wearable biosensors, in optical (colorimetric) wearable biosensors, usually only fluidics and sensing layers are needed, cutting the cost of electronics design and manufacturing. In one instance for nucleic acid detection through optical transduction mechanisms, after fabricating the microfluidic channels in an Ecoflex layer, Yang et al., attached the microfluidic on a bandage-like biosensor to assist with the adhesion of the Ecoflex layer to the user’s skin (Figure 2i) [30]. In another study, cellulose scaffolds prepared from filter paper were loaded with CRISPR-based wearable assay [25]. These scaffolds were then loaded into the prepared Ecoflex elastomer to make a wearable patch integrated with a cell-free synthetic biology-based assay. A colorimetric readout was observed upon detection. A recent study uses optical fluorescence intensities generated by the attachment of a particular biomarker to the aptamer-quencher complex to assess interstitial fluids utilizing aptamer-immobilized hydrogel microneedle patches [59]. To fabricate the microneedle patches, a mixture of methacrylated hyaluronic acid (MeHA), photoinitiator, and N,N′-methylenebisacrylamide (MBA) was prepared in buffer solution and applied to a negative PDMS mold. The hybridized aptamer-quencher complex was added to the mold before the previous layer was fully cured. To create the base of the patch, another layer of MeHA is added on top. However, the mold needed to be then washed to remove unbound aptamers and exposed to ultraviolet light for 5 min [59]. Microneedle-based wearable devices are new avenues for integrating nucleic acid-based assays. Nevertheless, careful assay design consideration for dynamic and real-time applications is crucial. These assays should be engineered in a way that would eliminate the need to use benchtop fluorescent or colorimetric instruments for quantitative analysis of the readout signal.

## 3. Biorecognition Requirements—Nucleic Acid-Based Assays

On the front line of the sensing layer of a wearable biosensor is the biorecognition element. Biorecognition elements are biomolecules that interact with the target analyte and determine crucial performance parameters, such as specificity and sensitivity. This biorecognition of an analyte is then translated to an interpretable signal by the sensing layer transducers [60]. The conventional recognition probes used in wearable biosensing research are antibodies and enzymes [13]. The antibodies are natural proteins with a “Y”-shaped 3D structure, which enables them to bind to their targets in a very specific and sensitive manner. Nevertheless, the discovery, isolation, and purification of antibodies are hindered by slow and expensive processes involving injecting antigens into laboratory animals, such as rabbits, mice, and goats [13,61,62]. Monoclonal antibodies are advantageous when it is necessary to ensure that target specifically binds to a specific location on the antibodies—a key to highly specific and sensitive detection. However, genetic mutation is risky when employing hybridomas to synthesize monoclonal antibodies. Since this genetic mutation may affect the antibody that the hybridoma produces, there may be variations over time across batches. Particularly, the specificity of the antibodies generated by the hybridoma may alter, and the paratope binding site may be affected by genetic alternation [63]. Additionally, the orientation of the target site on a functionalized antibody on a wearable biosensing platform is very important to the performance, as it could inhibit the target binding and reduce the sensitivity [64]. Enzymes are another class of naturally occurring proteins utilized as biorecognition elements in wearable biosensors [65,66]. Enzymes are catalytic proteins that have extensively been used in wearable biosensors due to their ability to be continuously used for a long time, ease of functionalization on the sensor, and specificity for the target analyte [64]. Currently, the most common enzyme synthesis for wearable biosensing applications is through the production of microbiological enzymes by fermentation under controlled circumstances. While working with enzymes, stability towards long-term application without losing their functionality due to degradation is the most crucial concern, forcing producers to stabilize the enzymes to increase the biosensor’s overall shelf life and operating stability. For example, alcohol oxidase is an enzyme used to detect sweat or interstitial alcohol concentrations when integrated with wearable biosensors [67]. The optimum pH of the functionalized alcohol dehydrogenase is basic (pH 8–10) [68]; however, the pH of the sweat and the interstitial fluid is slightly acidic to neutral [69]. This pH mismatch leads to a decrease in enzyme activity when used for wearable biosensing applications. Similarly, fluctuation in the bodily temperature may affect the enzyme activity by either affecting their activation energy or thermal stability. Another example is organophosphorus acid anhydrolase (OPAA), which is used to detect toxic organophosphorus compounds [70]. One of the main issues with OPAA is that their optimal operating temperature is 45 °C, which is significantly higher than the human body temperature, thus limiting their practical use in wearable biosensors. Additionally, the sample environment must be considered to sustain the enzyme’s optimal catalytic activity in biosensors. For instance, the enzyme glucose oxidase (GOx), is used in glucose wearable biosensors—one of the most clinically successful wearable biosensors to detect interstitial fluid glucose levels [71]. Generally, after binding to the substrates, enzymes catalytically convert them to reaction products, which can be measured on the sensing electrodes. During the sensing mechanism, the co-produced hydrogen peroxide for oxidase-based enzymes may weaken the enzyme structures. These enzymes are prone to deactivation if they are incorrectly applied during their application [18].

To develop clinically relevant wearable biosensors, it is of paramount importance to take advantage of biorecognition elements that are sensitive, specific, stable, consistent, and easy to produce [7]. Recently, the utilization of nucleic acids as biological recognition layers in wearable biosensors has taken momentum [72]. These simultaneously offer advantages unique to enzymes and antibodies by offering great versatility in customization and biological specificity [73]. NA production is an in vitro process that does not require any host animal for its production and can be rather created by adding mononucleotides to a 3′-hydroxyl end of the polynucleotide chain [20]. Consequently, their production takes less time, since it is not dependent on the immune system of a host animal, and it is easier to modify their structure without being concerned with issues such as genetic drifting [74]. The commonly used nucleic acids incorporated in wearable biosensors are deoxyribonucleic acid (DNA), ribonucleic acid (RNA), and aptamers [75] (Table 1). Moreover, in recent years, given the success of clustered regularly interspaced short palindromic repeats (CRISPR)-based systems for in vitro diagnostic platforms, their precision has been harnessed to detect sweat analytes in wearable biosensing technology as well [25,76].

### 3.1. Synthetic Oligonucleotides

**Figure 3:**
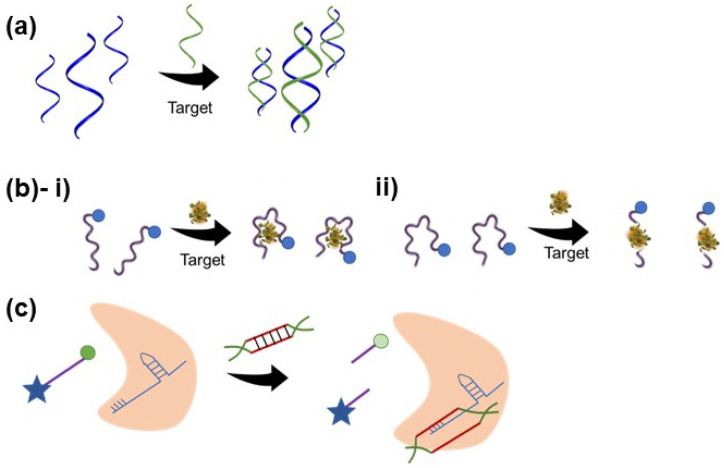
Design of assays for (**a**) synthetic oligonucleotides; target ssDNA hybridizes with the complementary sequence of the ssDNA probe and forms a nucleic acid duplex, (**b**) aptamers; upon binding to their target via intermolecular interactions, aptamers undergo a three-dimensional (**i**) signal on and (**ii**) signal off conformation rearrangement, and (**c**) CRISPR-Cas; Cas enzymes, upon binding to RNAs, form a complex which can search for and cleave target sequences complementary to the crRNAs.

Among various nucleic acids, synthetic oligonucleotides such as DNAs and RNAs are popular identification probes for detecting nucleic acid biomarkers [77]. Using a very straightforward approach, the single-stranded DNAs hybridize with the complementary sequence of the target DNA or RNA, thus forming a nucleic acid duplex (Figure 3a) [75]. This is also the case for RNA-based sensors, except RNA probes hybridize with RNA targets [75]. This mechanism has also been employed by Gao et al. to develop a wearable biosensor to detect a target miRNA sequence. To improve the detection limits of ssDNA or miRNA targets, they immobilized a dsDNA on the graphene surface. The 10 adenine bases at the 5′ end of the probe was added to enable their functionalization to the graphene sensing surface. The sequence from the middle to 3′ end was complementary to the target, miRNA-4484. The partial hybridization of probe-target, rather than complete hybridization, prevents nonspecific absorption of the target by graphene, enhancing sensitivity by reducing background signal. Their biorecognition probe achieved a detection limit of 10 fM within 20 min, at room temperature; this small sample incubation time further prevents probe desorption [46]. This was compared to another graphene-based biosensor with an ssDNA probe and an ssDNA target, with a detection limit of 1 pM [78]. The 100 times higher detection limit has been attributed to target absorption by the released dsDNA (hybridized probe and target) from the graphene surface rather than hybridizing with the capture probe [46]. As such, this hybridization-based sensing mechanism, and the fact that DNA and RNA sequences can be readily, and exactly constructed based on target complementarity, render good sensitivity and specificity for DNA/RNA-based biosensors [73]. However, this sensing mechanism limits the application of DNA and RNA probes to nucleic acid targets, and direct detection of other analytes, such as proteins, has not been reported.

**Table 1:** Nucleic acid-based wearable biosensors.

Sensing Material	Target Analyte	Transduction Method	Limit of Detection	Reference
**Synthetic nucleotide**				
**Ecoflex microfluidics**	Nucleic acid fragments of Zika virus	Fluorescence	10 copies μL^−1^ (1.66 × 10^−2^ fM)	[30]
**PDMS**	E. coli O157:H7 SARS-CoV-2	Colorimetry	500 pg/reaction (2.44 × 10^−1^ nM) 600 fg/reaction (3.03 × 10^−1^ pM)	[32]
**PDMS**	HIV-1 DNA	Fluorescence	100 copies mL^−1^ (1.66 × 10^−4^ fM)	[34]
**Graphene—based FET with PDMS microfluidics**	miRNA-4484	FET	10 fM	[46]
**Hydrogel microneedles modified with gold nanowires**	Epstein−Barr virus cell-free DNA	Electrochemical	3.7 × 10^2^ copies μL^−1^ (6.1 × 10^−1^ fM)	[50]
**CRISPR-Cas**				
**CRISPER-based Freeze-dried cell-free synthetic circuit**	MecA gene HIV RNA Ebola virus RNA	Fluorescence Colorimetry Luminescence	2.7 fM 10 μM 300 nM	[25]
**Aptamers**				
**In_2_O_3_ FET on polyamide with tape-based microfluidics**	Cortisol	FET	1 pM	[27]
**Gold modified electrodes with Graphene-gold nanoparticles with SU-8 microfluidics**	IL-6 IL-8 TGF–β1 Staphylococcus aureus	Electrochemical	10 ng mL^−1^ (4.76 × 10^−1^ nM) 10 ng mL^−1^ (1.18 nM) 50 pg mL^−1^ (1.13 pM) 1 ×10^8^ CFU mL^−1^	[29]
**Graphene—Based FET on ultrathin Mylar**	TNF-α IFN-γ	FET	2.75 pM 2.89 pM	[41]
**Graphen-Based FET on SiO_2_ Coated PEN**	TNF-α	FET	26 pM	[42]
**Platinum-Graphene Extended gate electrode**	Cortisol	FET	0.2 nM	[47]
**MeHA functionalized hydrogel microneedles**	Glucose ATP L-tyrosinamide thrombin	Optical	1.1 mM 0.1 mM 3.5 µM 25 nM	[59]
**Graphen-Nafion composite film**	IFN-γ	FET	740 fM	[79]
**ZnO on microporous hydrophilic membrane**	Cortisol	Electrochemical	0.11 µM	[80]
**ZnO coated nano-porous polyamide**	Cortisol	Electrochemical	2.7 nM	[81]
**PDMS@CNC/CNT**	Cortisol	Electrochemical	5 nM	[82]
**In_2_O_3_ nanoribbons on PET**	Serotonin Dopamine	FET	10 fM	[83]
**PEDOT-PAN nanofibers FET on PET**	Cortisol	FET	10 pM	[84]

One of the main aspects of DNAs- and RNAs-based sensing approach is their ability to integrate with the amplification, further improving the sensitivity [75]. Nucleic acid amplification methods, such as polymerase chain reaction (PCR), are currently widely used to detect nucleic acid materials in a benchtop setting. However, incorporating these methods into wearable biosensors requires circumventing the need for thermal cycling and a continuous heat supply. Isothermal nucleic acid amplification techniques are performed at lower temperatures than PCR and do not require thermal cycling hence providing point-of-care testing abilities. These techniques include Nucleic Acid Sequence-based Amplification [85], Loop-mediated Isothermal Amplification [86], Strand Displacement Amplification [87], Rolling Circle Amplification [88], and Recombinase Polymerase Amplification (RPA) [89]. RPA has the fastest reaction time, a near 37 °C reaction temperature, and has been employed in wearable devices, as it is highly sensitive, requires two specific forward and reverse primers, and can use human body heat as the heat source [46,76,77]. Trinh et al. developed a wearable RPA device, capable of forming contact with skin. Their device was successfully used to amplify 210 bp from Escherichia coli O157:H7 (*E. coli* O157:H7) and 203 bp from the DNA plasmid SARS-CoV-2 within 23 min using the heat generated by the human body. The limit of detection (LOD) for the genomic DNA template (*E. coli* O157:H7) was approximately 2.44 × 10^−1^ nM, and the plasmid DNA template (SARS-CoV-2) was 3.03 × 10^−1^ pM.

### 3.2. Aptamers

Among other nucleic acid-based biorecognition elements, aptamers are gaining much interest in developing wearable biosensors [25,27,29,41,42,47,79,80,81,82,83,84]. Aptamers are short single-stranded functional oligonucleotides (DNA or RNA, 10–60 nucleotides), capable of binding to cells, proteins, nucleic acids, ions, and virtually any target molecule [73,75]. Aptamers sequences are developed by systematic evolution of ligands by exponential enrichment (SELEX), an iterative and in vitro selection process that includes stepwise optimization of random sequence libraries until a high-affinity sequence for a specific target is reached [60]. This cost-effective process enables the design of an aptamer probe with high affinity and specificity for any given analyte, with maximal batch-to-batch consistency and without any prior knowledge of the target [73,80]. Moreover, they can be easily modified with different chemical moieties and can be integrated with various sensing mechanisms [73,80]. Unlike DNA/RNA-based sensors, which rely on partial or complete hybridization with the target, aptamers undergo a three-dimensional conformation rearrangement upon binding to their target via intermolecular interactions (Figure 3b) [60]. Thus, the aptamer-target interaction is similar to that of antibody-antigen. However, in the case of wearable biosensors, despite the high specificity of antibodies, aptamers are the preferred capture probes, as they are much smaller in size, and a high surface density can be achieved [80]. Additionally, upon association with the target, the distance between the target and the sensing platform might alternate, causing a stronger detection signal through their conformational change [42]. Moreover, wearable technologies often expose unfavorable temperatures and mechanical forces. Therefore, a key requirement is that the sensing probes must be resistant to structural alterations. Aptamers are chemically, thermally, and structurally more stable than their antibody counterparts [80,82]. Besides, the aptamer-target affinity is more stable throughout the storage period, granting aptamer-based wearable biosensors longer shelf lives [42,80]. Specifically, in wearable biosensing, aptamers are the most frequently used for quantifying cortisol concentrations in biological fluids, such as saliva or sweat [27,47,80,81,82,84]. In a recent study, Wang et al. discovered a novel DNA aptamer sequence for cortisol binding with a detection limit of 1 pM and a dissociation constant of 500 nM. The aptamer was discovered from an N36 random oligonucleotide library by SELEX based on the elution of cortisol-bound sequences versus capture strand binding. The aptamer could sense the physiologically relevant concentration of cortisol in saliva and sweat. Moreover, it was demonstrated that the sequence selectively binds to cortisol compared to non-target molecules with biological relevance to the target, such as corticosterone, testosterone, and aldosterone. These results, combined with the ability of the developed wearable biosensor to detect the fluctuations in the physiological range of cortisol concentration, suggest the translational potential of the sensor for the real-time monitoring of cortisol levels in saliva and sweat [27]. Other analytes detected by wearable aptamer-based biosensors using structure switching and surface charge alteration-based transduction include cytokines [29], serotonin [83], and dopamine [83]. Besides aptamers, other functional nucleic acids, such as DNAzymes [26] and ribozymes [90], also have promising potential to be integrated into wearable biosensing after showing successes in in-vitro and precision diagnostics areas. Moreover, as the binding of the aptamers to their targets is reversible, aptamer-based sensors can be regenerated and reused multiple times, leading to more cost-effective sensors [91]. The regeneration has been demonstrated by washing with a running buffer [92] or guanidine hydrochloride solution [93], incubation in 10% (*w*/*v*) sodium dodecyl sulfate [94], and introducing azobenzene moiety into the aptamer chain [95].

### 3.3. CRISPR-Cas

Recently, CRISPR-Cas technology has gained enormous attention as a highly precise gene editing tool. CRISPR, along with CRISPR-associated enzymes (Cas), composes an adaptive immune system mechanism against foreign genetic materials, found in microorganisms. These microorganisms can incorporate sequences of foreign nucleic acids into the CRISPR arrays on their genome, which are subsequently transcribed to give CRISPR RNAs (crRNA). Cas enzymes, upon binding to these crRNAs, form a complex which can search for and cleave target sequences complementary to the crRNAs (Figure 3c) [96]. The advantages of such a system include its inherent high sensitivity (detection limits as low as ~50 fM, as reported in the literature) [97], due to Cas enzymes’ specific sequence requirements for cleavage, leading to resolution down to a single base pair, low development cost, rapid reaction time (0.5–2 h), and programmability to target any nucleic acid sequence or protein sequences if coupled with aptamer/DNAzyme-based assays [25,51,98]. However, CRISPR-based biosensors have been integrated with mainly fluorescent transduction and lateral flow strips, which sometimes rely on heavy instruments, and have low sensitivity [76]. Additionally, for low target concentrations, CRISPR-based biosensors are usually coupled with a nucleic acid amplification method to augment signal strength, making portability a challenge [51]. As discussed earlier, since PCR requires specific equipment and professional operators, isothermal nucleic acid amplification methods may be used with CRISPR-based sensing devices. Successful attempts have been made to develop amplification-free CRISPR-based assays, by using gold nanostructures [51]. In the context of wearable biosensors, Nguyen et al. recently used a CRISPR system as an unlocker, which activates a subsequent recognition element in their sensing device. Upon binding the Cas12a-guide RNA complex to the target dsDNAs, resistance-associated genes of Staphylococcus aureus (mecA, spa, and ermA), the complex cleaves a quenched ssDNA fluorophore probe, generating a fluorescence signal. By coupling their system with RPA, they managed to harness the unique features of CRISPR-based systems and improve signal transduction to detect 2.7 fM of mecA gene. Moreover, such a system can be freeze-dried to give stable products that can be activated by hydration [25]. Introducing this strategy to other nucleic acid-based sensors can further enhance the stability and shelf-life of the sensors.

For the next generation of wearable biosensors, some challenges remain in providing robust continuous monitoring of health status at the molecular level. While antibodies and enzymes pose limitations in stability and are only limited to longitudinal (discreetly over a period of time) detection of biomarkers, nucleic acids, and specifically, functional nucleic acids such as aptamers, DNAzymes, and other cell-free synthetic biology-based assays, provide an opportunity for real-time continuous monitoring due to the capability of reagent integration, stability, and regeneration [99]. On the other hand, other cell-free synthetic biology-integrated wearable biosensors are being developed, though it is not yet understood how feasible they are in terms of the long-term stability of the reagents.

## 4. Transduction Mechanism Requirements

**Figure 4:**
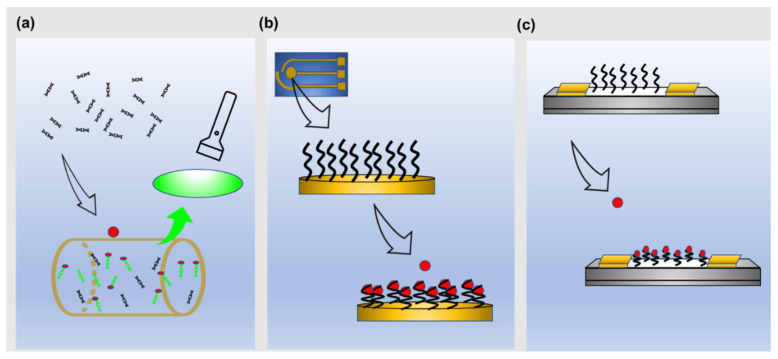
Schematic illustration of various transduction mechanisms, i.e., (**a**) optical, (**b**) electrochemical, and (**c**) electrical.

A transducer on a wearable biosensor converts the biorecognition event into a readable signal and clinically relevant information via different physical methods. Popularly the transduction methods in a wearable biosensor can be categorized into optical, electrical, and electrochemical (Figure 4a–c) [29,30,81]. These methods provide opportunities for facile operation via miniaturization, prompt measurements, and low operating power [81,100,101,102]. The biochemical signal produces during the interaction of the biorecognition element (NA), and the target analyte is often very low. Hence, a good signal transduction strategy becomes extremely important for translating this low signal to an amplified strength with a high signal-to-noise ratio [103].

### 4.1. Optical

**Figure 5:**
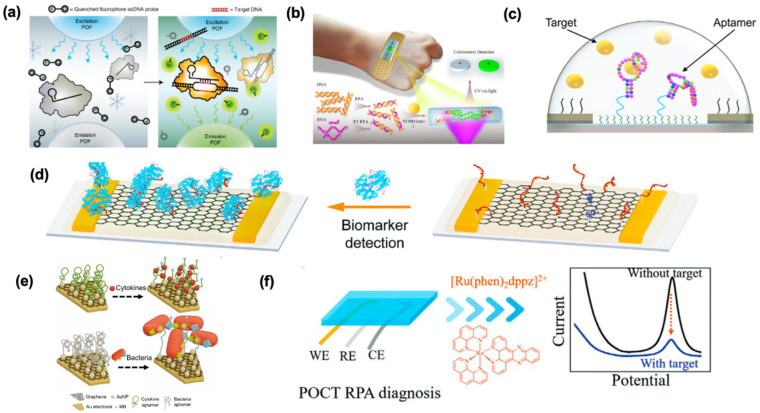
Sensing mechanisms of optical, electrochemical, and FET-based wearable biosensor. (**a**) When the FDCF wearable system is rehydrated containing the mecA gene, generated RPA amplicons activate the Cas12a complex. The quenched ssDNA fluorophore probe is then cleaved, producing a fluorescence output (reprinted with permission from Refs. [65,66] Copyright 2021, Nature). (**b**) When nucleic acid fragment of zika virus is present, SYBR green 1 interacts with RPA double stranded amplicon to produce a fluorescence readout (reprinted with permission from Ref. [27] Copyright 2019, Elsevier). (**c**) Aptamer binding under the presence of cortisol induces conformational changes to the aptamer-cortisol complex and changes in electrical signals are subsequently measured (reprinted with permission from Ref. [22] Copyright 2022, the authors). (**d**) In the presence of IFN- γ, the aptamer changes conformation and moves closer to the sensing channel, creating a change in signal (reprinted with permission from Ref. [80] Copyright 2021, John Wiley and Sons). (**e**) The redox probe moves further away from the electrodes when cytokine and S. aureus are present, thereby decreasing the current (reprinted with permission from Ref. [23] Copyright 2021, the authors). (**f**) In the presence of the target DNA, RPA amplification occurs, [Ru(phen)2dppz]BF4 detects double stranded amplicons, and a decrease in redox peak current is observed (reprinted with permission from Ref. [104] Copyright 2020, John Wiley and Sons).

Majority of nucleic acid assay integrated wearable biosensors rely on optical methods for biosensing [101] where an optical transducer measures the change in optical properties to create a signal output in the form of colorimetry, fluorescence, and luminescence, to name a few (Figure 4a) [25,30,34]. Nguyen et al. investigated an extensive list of innovative wearable biosensors using freeze-dried cell-free (FDCF) synthetic circuits to optically (colorimetric, fluorescence, and luminesce) detect infectious target molecules, such as a SARS-CoV-2 gene region, Ebola virus RNA, and HIV virus RNA. Freeze-dried cell-free synthetic circuits are free-standing abiotic systems with all biomolecules needed to activate biological machinery, such as transcription and translation, whereby genetically engineered circuits with DNA or RNA can be incorporated [25]. These circuits are activated by rehydration. For example, Nguyen et al. designed CRISPR-Cas 12a complexes with recombinase polymerase amplification (RPA) to detect the mecA gene (Figure 5a), which is commonly present in methicillin-resistant Staphylococcus aureus [25]. In this design, the integration of CRISPR-based FDCF sensor circuit, polymeric optic fibers, and spectrometer into a garment was created to continuously monitor fluorescence output. The wearable spectrometer detection system is connected to a wireless application for convenient and continuous monitoring using a smartphone. This kind of sensor integration with textile requires careful consideration of the material used for fabrication so that signal mismatch can be avoided. The FDCF wearable system is rehydrated and activated by contaminated splashes containing the mec A gene as it wicks through the device [25]. These contaminated flashes were extracted from the human participants’ sweat during exercise. In the presence of the target contaminated fluid, Cas 12a detected RNA amplicons of the target DNA sequence through guide RNAs, and its non-specific cleaving activity was activated. Subsequently, trans-cleavage of quenched ssDNA fluorophore probe occurs and was detected as a fluorescence output in 90 min with a detection limit of 2.7 fM. In a similar textile FDCF technology platform, a slightly different transduction mechanism was employed. Specifically, a toehold switch with an integrated nanoluciferase operon was used to detect HIV RNA by generating a luminescence signal. In this design, the expression of the reporter gene is repressed until binding between the target RNA and the sensing domain of the toehold switch occur. Specifically, when the HIV RNA binds with the sequence in the sensing module, the ribosome binding site, and start codon are activated, and translation of the non-luciferase operon occurs by which its gene product is utilized as a luminescent reporter. Using the luminescence method, 10 μM of HIV RNA target was detected. Nguyen et al. also developed a stand-alone colorimetric detection platform for the Ebola virus RNA. In this case, a lacZ β-galactosidase operon circuit with a toehold switch was merged in a cellulose substrate, and layers of flexible elastomers were used for assembly. This assembly was successfully implemented into a bracelet. Akin to the mechanism described above, when the Ebola virus RNA is present in the contaminate splash, the sensor is activated. Hybridization upstream of the toehold switch then occurs, allowing for the expression of the lacZ gene. Integrated within the device, chlorophenol red-β-d-galactopyranoside is then hydrolyzed, and a color change from yellow to purple is made visible. After 30 min, detection of the 300 nM of Ebola virus RNA was observed. Colorimetric methods are more facile than fluorescence and luminescence methods, since there is no need for signal interrogation using a physical device, and the output signal can be easily distinguished in visible light. Conversely, fluorescence and luminescence methods used in this paper required the employment of an on-body fiber optic network and a spectrometer to interrogate and then analyze the signal through a wireless mobile system. It is important to note that in all of Nguyen et al.’s work, the in-garment sensors were only tested on a mannequin and that biological fluids used as a sample were synthetically made. Improvement for these devices would be to test on human participants. The fluorescence method used in this study had the lowest limit of detection in the femtomolar range, while the luminescence method had the highest limit of detection in the micromolar range. Moreover, visible fluorescence or luminesce was evident 5–20 min after exposure, while visible changes occurred after 40–60 min for the colorimetric method [25]. A strict calibration for quantitative analysis is a key requirement as the qualitative outcome of these methods is difficult to differentiate between varying concentrations of the target analyte. The same was demonstrated experimentally in this work as well.

A novel fluorescence assay utilizing aptamer onto cross-linked methacrylated hyaluronic acid hydrogel microneedle patch biosensor enabled rapid detection of many biomolecules, such as glucose, adenosine triphosphate (ATP), L-tryosinamide, and thrombin. The transduction mechanism used in this assay is unique in that it allows for on-needle signal detection. Upon minimally invasive insertion of the microneedles into the skin, interstitial fluid (ISF) was sufficiently extracted. The aptamer is conjugated with a Cy3-fluorophore and hybridized with a quencher-conjugated DNA competitor strand, which serves to decrease the intensity of the fluorescence. A reagentless approach is used where in the absence of the target in the extracted ISF, the quencher and fluorophore are in proximity, and no fluorescence output is observed. During target binding, the competitor strand along with the quencher dissociates from the fluorophore-conjugated aptamer, and fluorescence is observed. The detection limit was 1.1 mM, 0.1 mM, 3.5 uM, and 25 mM for glucose, ATP, L-tryosinamide, and thrombin, respectively, and showed high selectivity against nonspecific targets. The biosensor patch was also successfully applied to the skin of diabetic rat models. A key drawback is that fluorescence was recorded using a bench top fluorescence microscope, which takes away the ease-of-use characteristic of wearable platforms. Continuous measurement can be achieved by using a linker that prevents complete removal of the quencher-conjugated DNA from the aptamer upon target binding. Miniaturization of optical detecting strategies would also be an improvement for this device [59]. Another study developed a body heat-activated wearable bandage sensor for the detection of nucleic acid fragments of the zika virus by combining flexible microfluidic technology and RPA [30]. This biosensor highlights the achievability of on-body signal transduction since body heat was needed to activate the reaction. In principle, when the zika-specific DNA fragments are present along with the target specific RPA reagents in the microchip, exponential amplification of the fragments through RPA are done [30,34]. The SYBR green 1 in the reaction reservoir interacted with the RPA double stranded amplicons to trigger fluorescence. After the sensor was incubated on human wrist for a reaction to occur, fluorescence was observed using a UV-torch for naked-eye detection, which again could be improved by miniaturization of the optical measuring device (Figure 5b) [30]. While the wearable biosensor demonstrated significant change in fluorescence with exposure to target DNA, no significant changes in fluorescence was observed in the fragment DNA of West Nile virus, Dengue virus, and Japanese encephalitis virus. In 10 min, the sensor was able to detect target DNA with a limit of detection of 10 copies/uL. Reproducibility was shown when fluorescence intensity across different target concentrations for five similar sensors were compared. Two drawbacks of the optical method for biosensing are made clear in the Nguyen et al.’s work. First, quantitative detection of the target was not possible because color intensity did not accurately reveal target concentration. This could be solved by an electro-optical microchip as part of the assembly [30] Next, nucleic acid detection of this device requires non-automated sampling processes. Since obtaining biofluids and sample pretreatment should all be integrated within the device, this device lacks the convenient characteristics of wearable biosensors. A similar strategy was developed by Trinh and Lee for detecting *Escherichia coli* (*E. coli*) O157:H7 and DNA plasmid of SARS-CoV-2 using a wearable PDMS microdevice along with the RPA technique operated by body heat. The wearable device was able to detect 500 pg/reaction of genomic DNA of *E. coli* O157:H7 and 600 fg/reaction of plasmid DNA of SARS-CoV-2 [32]. In addition, HIV DNA as low as 100 copies/mL was also detected using the same sensing technique [34].

Note that the sensitivity of the RPA method integrated into these wearable devices is up to par with the polymerase chain reaction method, indicating that sensitivity is not compromised for simpler diagnostic testing [30,32]. In addition, the optical wearable biosensors enable ease of use where qualitative can be easily interpreted by untrained personnel. However, the main disadvantage that limits optical signaling methods in wearable biosensing scheme is its complicated qualitative to quantitative data conversion, thereby reducing its potential to be integrated into wearable biosensors for in-depth and real-time monitoring and analyses.

### 4.2. Electrical

The electrical transduction mechanism for nucleic acid integrated wearable biosensors is dominated by field effect transistor (FET)-based biosensors. FETs are semiconducting devices consisting of three electrodes, namely, a source, a drain, and a gate. The biorecognition elements are immobilized mainly on the gate [105]. Detection of the target of interest is achieved by target binding induced changes in the conductivity of the semiconductor channels, of which quantitative electrical measurements are obtained [105]. FETs show phenomenal sensitivity, with limits of detection moving into the zeptomolar range and capabilities to detect single molecules [47,102,106]. However, the Debye length limits the sensitivity of receptor-modified FETs [107]. Specifically, the intermolecular repulsive forces between the nucleic acid targets are shielded by the high ionic strength of physiological conditions of the solution [104]. High ionic strengths of the electrolyte lead to a short Debye length, which is the electrostatic screening effect of charges in solution. This limits the critical length of the probe immobilized on FETs, since charges are electrically screened pass the Debye length, which is measured starting from the electrode [104,107,108]. In physiological conditions, the critical Debye length is less than 1 nm; hence, incorporation of conformation-changing nucleic acids upon target recognition enable a significant signal because structural changes are likely to occur within the Debye length and can generate sufficient electric changes [47,102,105,107]. Recent advances in developing FET wearable biosensors have been made, one of the most explored NA-based strategy being the development of cortisol detecting FETs with aptamers immobilized on of different novel material such as ln_2_O_3_ thin nanofilms sensing channels [27]. Interestingly, the cortisol sensing array was assembled into a watch, where it was able to both collect and analyze real-time cortisol levels in sweat at single points in time. When cortisol interacts with the aptamer, conformational changes of the phosphodiester backbones of the aptamers cause surface charge perturbations and are measured as electrical signals in correlation with varying cortisol concentrations (Figure 5c) [27]. When tested using artificial sweat, the limit of detection of 1 pM with a detection range of 1 pM to 1 μM was obtained for this biosensor. The biosensor demonstrated high specificity as insignificant responses were measured against non-relevant targets like testosterone, progesterone, corticosterone, and aldosteronein. The device was also tested in saliva samples from participants in a Tier and Social Stress Test and measurements were consistent with the cortisol level trends obtained from standard laboratory assays. Moreover, the device was also used to determine cortisol level changes according to the circadian rhythm and showed consistent results from ELISA analysis of the samples used. Before performing on-body sweat measurements, the assembled device was attached to the wrist skin of a healthy person with double-sided tape, and FET sensor baselines were recorded in artificial sweat for calibration. The target stimulation region of the skin was first cleansed with deionized water and ethanol prior to five minutes of iontophoretic sweat gland stimulation. Precipitation is then transferred from the skin to the sensor array with the help of the microfluidic layer. Akin to the development described above, several other studies have also investigated the use of FET as the sensing strategy, in attempts to detect cortisol at physiologically relevant concentrations using aptamer-cortisol binding, which enabled signal change. For instance, an investigation used a liquid-ion gated field effect transistor consisting of an aptamer conjugated poly(3,4-ethylenedioxythiophene) polyacrylonitrile nanofibers (PEDOT-PAN NFs) as its sensor channel [84]. The PEDOT-PAN NFs FET biosensor had a limit of detection of 10 pM of cortisol with a range of response between 1 pM to 10 μM. To test the wearable applicability of the device, the sensor was placed on human skin while participants exercised. In this test, the wearable sensor was able to detect and discriminate between various levels of cortisol from human sweat measured at different times of day. The aptamer-functionalized graphene layer on a platinum gate electrode of an extended field effect transistor to detect cortisol was also investigated. The device had a detection limit of 0.2 nM and a detection range between 1 nM and 10 μM [47]. While quite efficient and sensitive in their application for cortisol detection, this research has not utilized the full potential of aptamer chemistry and regeneration for continuous long-term use.

Additionally, one study aimed to detect IFN-γ, a cytokine biomarker (Figure 5d), by developing a FET flexible biosensor with a regenerative aptamer-functionalized graphene-Nafion composite film as its sensing channel [79]. The negatively charged IFN-γ-aptamer complex and its compact conformation moves close to the sensing channel and induces a change in current detected upon target binding. The limit of detection was 740 fM in undiluted human sweat and 880 fM when mounted onto an artificial hand. At equal concentrations, the change in signal for IFN-γ is five times greater in comparison to non-specific targets, such as TFN-a, interleukin-002, and interleuk-6. After biomarker detection, the biosensor was dipped in ethanol to dissolve the Nafion film and displace the bound aptamers. Nafion and aptamers were then drop-casted and functionalized, respectively, back to the FET sensor. Regeneration was successful for 80 cycles, with sensor response consistent with the first cycle. Previously, Z. Wang et al. developed a wearable graphene-based FET functionalized with aptamers for detection of cytokines including IFN-γ, which demonstrated a limit of detection of 2.89 pM [41]. This new finding indicates an improvement in design and development of wearable biosensors for detecting IFN-γ with FET as its sensing component. Multiplex analysis of biomarkers using FET has also been accomplished and offer more information on one’s health. For instance, a multiplexed ln_2_O_3_ nanoribbon FET functionalized with aptamers to detect serotonin and dopamine was developed with a limit of detection of 10 fM and a detection range between 0.1 pM and 1 μM [83].

### 4.3. Electrochemical

Electrochemical-based wearable biosensors are coming out to be most promising given their advantages, i.e., reagent integrated analysis, sensitivity, and ease of fabrication. An electrochemical sensor integrates an electrochemical transducing element and immobilized bio-recognition elements capable of binding with a target [81,109]. As a result of target binding, the chemical reaction is transduced into an electrical signal that can be measured corresponding to target concentrations [109]. Along with these attractive features come some challenges, including sensitivity to sample matrix effects, narrow optimal temperature range for operation, and restrictive shelf life [110,111]. Continuing with the trend of aptamer-based wearable biosensors for sweat cortisol detection, a watch, named CATCH, consists of an aptamer-functionalized ZnO coated sensor surface with a nano-porous polyamide substrate to detect cortisol [81]. Specifically, target binding causes an increase in charge transfer resistance and, using electrochemical impedimetric spectroscopy, concentration-dependent responses are quantitatively measured. As an alternative technique, chronoamperometry was also utilized to confirm the performance of the sensing platform. The sensor’s limit of detection was 1 ng/mL with a detection range of 1–256 ng/mL. Since cortisol levels fluctuate in accordance with the circadian rhythm, the aptasensor was able to capture the increasing and decreasing levels of cortisol. Aptamer-based sensors often require chemical reagents for regeneration of the device, and this study addressed this limitation by modifying their analytical strategy. They measured changes in cortisol concentrations from the change in slope of the previous concentration, where a slope change indicated a change in impendence response. This served as a novel way of extending on-body operation of the device. Whereas the sensor showed significant response to cortisol, insignificant signal responses were measured for non-targets at 200 ng/mL concentrations. To further cement its performance characteristics, the device was worn and used by healthy human participants and detected cortisol ranges from 1–12 ng/mL in sweat. Similar studies focusing on electrochemical-based sensors for cortisol detection in wearable formats were published around the same time. Ganguly et.al. demonstrated a limit of detection of 4 ng/mL, while in the work by Mugo et al., their device showed a limit of detection of about 1.8 ng/mL [80].

Another unique electrochemical-based flexible immunosensor with functionalized aptamers on graphene-gold nanoparticles nanocomposite modified electrodes for simultaneous detection of TNF-α, IL-6, IL-8, TGF-1, S. aureus, pH, and temperature was developed, which allowed monitoring of wound conditions [29]. Known as the VeCare platform, the biosensor that can be directly applied to a wounded skin incorporates a wound contact layer, a microfluidic collector that can collect target analytes, and a breathable barrier in the sensor complex. Without the analytes, the redox probe of the modified bound aptamers is close to the electrodes, paving the way for charge transfer. Following target binding, conformational changes of the aptamers move the redox probe further from the electrode and consequently reduces the redox current. A portable wireless analyzer was integrated in the sensor, which could perform and measure electrochemical tests. Data were wirelessly transmitted to a mobile device. Interestingly, the wireless analyzer was accompanied with a clinical management system, allowing for prompt clinical care in diagnostic and treatment (Figure 5e). All in all, this sensor design allowed for on body sampling, reagentless transduction, and analysis. The sensor demonstrated detection of target concentrations within physiologically relevant ranges through square wave voltammetry, selectivity against similar non-targets, specificity, and reproducibility. Moreover, clinical application of VeCare was also evident from correct analyses of wound exudate obtained from patients with venous ulcers [29]. Moreover, in situ wound monitoring was also tested in mice models, highlighting the potential of wearable applicability of the device.

Yang et al. also developed a wearable patch (Figure 5f) integrated with microneedles and reverse iontophoresis for in situ capturing of Epstein–Barr virus-free DNA from interstitial fluid, as well as an RPA electrochemical system for subsequent quantification of the target [112]. In the presence of the target DNA, RPA amplification occurs, activated by human body heat, and [Ru(phen)_2_dppz]BF_4_ is inserted in the minor groove of the RPA amplicons. The presence of the redox probe led to a decrease in redox peak current, and quantitative detection of the target DNA was obtained. Through this sensing mechanism, the detection limit was 1.82 × 10^−3^ fM [112].

Among many transducing mechanisms, optical-, electrical-, and electrochemical-based methods have shown promising results and are successfully integrated into wearable devices. Although these transducing methods offer many common advantages, the choice of the most appropriate sensing platform integrated into wearable devices relies on the clinical decision-making of the developed biosensor, whether to measure targets quantitatively or quantitatively. The majority of the wearable biosensing platforms discussed here utilized sample collection from the human participants (ISF, sweat, etc.) to demonstrate the validity of their wearable biosensing assays. Specifically, while detecting target analyte in ISF, it becomes increasingly difficult to perform detection while the device is inserted into the skin [113]. For sweat analysis, it becomes much easier to perform detection while the device is worn [113,114]. However, it requires that either the device is worn in places where the sweat is generated continuously, or the sweat is generated artificially by processes such as iontophoresis [115]. At this time, improvements on sensing platforms are being made to be used in clinical applications in the future.

## 5. Summary and Future Outlook

Medical wearable technology is becoming omnipresent with their use in not just monitoring physical and electrophysiological attributes of the human body but also by bringing on-body sensors to precision diagnostics field. This has led to intensive research in discovering non- or minimally invasive biomarkers (in sweat, tear, saliva, interstitial fluid) and implementation of new biorecognition elements, such as nucleic acids, to wearable motifs. Modern nucleic acid-based wearable devices incorporate three key components, namely biorecognition design, transduction mechanisms, and fabrication processes, to achieve high-precision continuous monitoring to further advance simplicity, sensitivity, specificity, and affordability. Microfluidic layers necessary for the collection of biomarkers, sampling, and reagent storage for nucleic acid-based assays are commonly fabricated using soft lithography or mechanical microfabrication techniques using polymers. A recent study showed a technique outside the aforementioned for double-layer PMMA mold development, specifically attaching the supporting microfluidic structure with a double-sided adhesive and utilizing it as a cast for another PDMS-based component [34]. Despite their high precision, flexibility, and toughness, the development of polymer-based microfluidics layers requires the use of non-biodegradable polymer supporting substrates and expensive fabrication infrastructure, as they are heavily reliant on clean room-based fabrication techniques. Polymer properties, however, can be harnessed to expand their use in wearable sensors such as liquid crystal polymer, stimulus response polymer gels, and piezoelectric polymers [116]. An emerging alternative to polymers is paper which has proven to be intrinsically sustainable, breathable, flexible, biocompatible, and biodegradable with potential for commercial translation. The trade off, however, is reduced patterning resolutions in comparison to other advanced fabrication techniques (soft lithography), but it can nevertheless serve as a valuable complement to existing fabrication methods of epidermal electronic devices. Sensing components are fabricated using various techniques that include a combination of lithography and deposition methods, such as E-beam evaporation, sputtering, or inkjet printing, such as conductive electrodes mounted on top of other polymers or integrated within the microfluidic layer. The functionalization of the biorecognition element on the sensing layer is also one of the important considerations while choosing the sensing layer material. Additionally, the readout or packaging layer currently limits the use of such wearable devices to a few hours in a day. While many power generation strategies exist that are attached with the readout layer, it is necessary to make these power generation units sweat resistant either by utilizing unique hydrophobic packaging materials or using sweat based power generation units [115]. In terms of biorecognition elements, nucleic acids as biological recognition layers offer greater versatility in terms of customization and biological specificity. Aptamer biorecognition elements, and other pseudo-natural modalities, provide a much wider range of biosensing applications with the ability to target various bio-analytes, including metal ions, small molecules, and more complex targets, such as whole cells. To date, wearable biosensors are based on antibodies and enzyme immobilized assays. Wearable biosensors integrated with an antibody assay detecting cortisol, for instance, one of the most studied sweat analytes, have demonstrated lower limits of detection compared with available nucleic acid-based sensors [117]. This non-invasive sensing approach is characterized by high performance attributed to the sensitivity of aptamer-based assays to transduce the antibody–cortisol interaction. Aptamer assays can provide high target binding density and loading density, as well as conformational change-based regeneration that can readily be used to amplify the transducing signal for wearable biosensing scheme. Additionally, some commercial wearable glucose monitors require invasive methods to detect the glucose levels in patients [17]. While they have quick readouts during their operation because of their enzymatic nature, users might find it difficult to attach these monitors to their body for long periods of time. There have been a lot of efforts in finding unique ways to do this continuous detection of glucose non-invasively. For example, glucose-specific aptamers can be immobilized on a sensing platform, while the sample fluids can be achieved from the patient’s sweat through microfluidics systems [107]. This design would be of benefit, both by eliminating the presence of hydrogen peroxide reactants and expanding usage of non-invasive wearable technologies—two critical considerations in current wearable glucose biosensing research. However, the practicality and long-term application of these concepts require more rigorous research in eliminating biofouling in sweat, background signal, and assay design. Even though they are more stable than their antibody and enzymatic counter parts, the desorption of nucleic acids from the sensing platform and their signal loss are still being investigated [118,119]. Another future direction would be to investigate other biorecognition regeneration processes, such as optical or electrical stimulation, to enable long-term usage on these wearable biosensing devices for chronic disease diagnosis applications. Various transduction mechanisms are discussed that include optical, electrical, and electrochemical methods, highlighting the wide use of optical properties for signal output. The disadvantage with optical sensing, however, is quantifying detection of the target, as color intensity does not accurately reveal target concentration. A possible solution to this challenge is the use of an electro-optical microchip for better quantification of target concentration. The choice of transduction method is largely dependent on the requirements of the clinical application, i.e., measuring targets either quantitatively or quantitatively. Integrating the wearable biosensors with machine learning, internet of things, and communication will also provide additional functionality that will enable actionable insights to the user and the healthcare workers. Future work on wearable technology should also take into consideration cost-effective fabrication, breathability of the devices, biodegradable or “green” materials, robustness to withstand motion induced artefacts, and improved scalability.

## References

[1] Wood C.S., Thomas M.R., Budd J., Mashamba-Thompson T.P., Herbst K., Pillay D., Peeling R.W., Johnson A.M., McKendry R.A., Stevens M.M. (2019). Taking Connected Mobile-Health Diagnostics of Infectious Diseases to the Field. Nature.

[2] Jeong J.-W., Yeo W.-H., Akhtar A., Norton J.J.S., Kwack Y.-J., Li S., Jung S.-Y., Su Y., Lee W., Xia J. (2013). Materials and Optimized Designs for Human-Machine Interfaces Via Epidermal Electronics. Adv. Mater..

[3] Pusta A., Tertiș M., Cristea C., Mirel S. (2021). Wearable Sensors for the Detection of Biomarkers for Wound Infection. Biosensors.

[4] Davies E.H., Johnston J., Toro C., Tifft C.J. (2020). A Feasibility Study of MHealth and Wearable Technology in Late Onset GM2 Gangliosidosis (Tay-Sachs and Sandhoff Disease). Orphanet J. Rare Dis..

[5] Cappon G., Acciaroli G., Vettoretti M., Facchinetti A., Sparacino G. (2017). Wearable Continuous Glucose Monitoring Sensors: A Revolution in Diabetes Treatment. Electronics.

[6] Smith E.N., Santoro E., Moraveji N., Susi M., Crum A.J. (2020). Integrating Wearables in Stress Management Interventions: Promising Evidence from a Randomized Trial. Int. J. Stress Manag..

[7] Kim J., Campbell A.S., de Ávila B.E.-F., Wang J. (2019). Wearable Biosensors for Healthcare Monitoring. Nat. Biotechnol..

[8] Bhide A., Ganguly A., Parupudi T., Ramasamy M., Muthukumar S., Prasad S. (2021). Next-Generation Continuous Metabolite Sensing toward Emerging Sensor Needs. ACS Omega.

[9] Mitra P., Sharma P. (2021). POCT in Developing Countries. EJIFCC.

[10] Slade Shantz J.A., Veillette C.J.H. (2014). The Application of Wearable Technology in Surgery: Ensuring the Positive Impact of the Wearable Revolution on Surgical Patients. Front. Surg..

[11] Weizman Y., Tan A.M., Fuss F.K. (2020). Use of Wearable Technology to Enhance Response to the Coronavirus (COVID-19) Pandemic. Public Health.

[12] Seshadri D.R., Li R.T., Voos J.E., Rowbottom J.R., Alfes C.M., Zorman C.A., Drummond C.K. (2019). Wearable Sensors for Monitoring the Internal and External Workload of the Athlete. NPJ Digit. Med..

[13] Tu J., Torrente-Rodríguez R.M., Wang M., Gao W. (2020). The Era of Digital Health: A Review of Portable and Wearable Affinity Biosensors. Adv. Funct. Mater..

[14] Sharma A., Badea M., Tiwari S., Marty J.L. (2021). Wearable Biosensors: An Alternative and Practical Approach in Healthcare and Disease Monitoring. Molecules.

[15] Windmiller J.R., Wang J. (2013). Wearable Electrochemical Sensors and Biosensors: A Review. Electroanalysis.

[16] AJ Bandodkar I.J.J.W. (2016). Wearable Chemical Sensors: Present Challenges and Future Prospects. ACS Sens..

[17] Lee H., Hong Y.J., Baik S., Hyeon T., Kim D.H. (2018). Enzyme-Based Glucose Sensor: From Invasive to Wearable Device. Adv. Healthc. Mater..

[18] Sonawane A., Manickam P., Bhansali S. (2017). Stability of Enzymatic Biosensors for Wearable Applications. IEEE Rev. Biomed. Eng..

[19] Micura R., Höbartner C. (2020). Fundamental Studies of Functional Nucleic Acids: Aptamers, Riboswitches, Ribozymes and DNAzymes. Chem. Soc. Rev..

[20] Khorana H.G. (1968). Nucleic Acid Synthesis. Pure Appl. Chem..

[21] Liu J., Cao Z., Lu Y. (2009). Functional Nucleic Acid Sensors. Chem. Rev..

[22] Famulok M., Hartig J.S., Mayer G. (2007). Functional Aptamers and Aptazymes in Biotechnology, Diagnostics, and Therapy. Chem. Rev..

[23] Paul R., Ostermann E., Wei Q. (2020). Advances in Point-of-Care Nucleic Acid Extraction Technologies for Rapid Diagnosis of Human and Plant Diseases. Biosens. Bioelectron..

[24] Gong L., Zhao Z., Lv Y.-F., Huan S.-Y., Fu T., Zhang X.-B., Shen G.-L., Yu R.-Q. (2015). DNAzyme-Based Biosensors and Nanodevices. Chem. Commun..

[25] Nguyen P.Q., Soenksen L.R., Donghia N.M., Angenent-Mari N.M., de Puig H., Huang A., Lee R., Slomovic S., Galbersanini T., Lansberry G. (2021). Wearable Materials with Embedded Synthetic Biology Sensors for Biomolecule Detection. Nat. Biotechnol..

[26] Pandey R., Chang D., Smieja M., Hoare T., Li Y., Soleymani L. (2021). Integrating Programmable DNAzymes with Electrical Readout for Rapid and Culture-Free Bacterial Detection Using a Handheld Platform. Nat. Chem..

[27] Wang B., Zhao C., Wang Z., Yang K.-A., Cheng X., Liu W., Yu W., Lin S., Zhao Y., Cheung K.M. (2022). Wearable aptamer-field-effect transistor sensing system for noninvasive cortisol monitoring. Sci. Adv..

[28] Takaloo S., Moghimi Zand M. (2021). Wearable Electrochemical Flexible Biosensors: With the Focus on Affinity Biosensors. Sens. Biosens. Res..

[29] Gao Y., Nguyen D.T., Yeo T., bin Lim S., Xian Tan W., Edward Madden L., Jin L., Yong Kenan Long J., Abu Bakar Aloweni F., Jia Angela Liew Y. (2021). A Flexible Multiplexed Immunosensor for Point-of-Care in Situ Wound Monitoring. Sci. Adv..

[30] Yang B., Kong J., Fang X. (2019). Bandage-like Wearable Flexible Microfluidic Recombinase Polymerase Amplification Sensor for the Rapid Visual Detection of Nucleic Acids. Talanta.

[31] Li X., Zhao X., Yang W., Xu F., Chen B., Peng J., Huang J., Mi S. (2021). Stretch-Driven Microfluidic Chip for Nucleic Acid Detection. Biotechnol. Bioeng..

[32] Trinh K.T.L., Lee N.Y. (2022). Fabrication of Wearable PDMS Device for Rapid Detection of Nucleic Acids via Recombinase Polymerase Amplification Operated by Human Body Heat. Biosensors.

[33] Bartholomeusz D.A., Boutte R.W., Andrade J.D. (2005). Xurography: Rapid Prototyping of Microstructures Using a Cutting Plotter. J. Microelectromech. Syst..

[34] Kong M., Li Z., Wu J., Hu J., Sheng Y., Wu D., Lin Y., Li M., Wang X., Wang S. (2019). A Wearable Microfluidic Device for Rapid Detection of HIV-1 DNA Using Recombinase Polymerase Amplification. Talanta.

[35] Xu Y., Zhao G., Zhu L., Fei Q., Zhang Z., Chen Z., An F., Chen Y., Ling Y., Guo P. (2020). Pencil-Paper on-Skin Electronics. Proc. Natl. Acad. Sci. USA.

[36] Xu Y., Fei Q., Page M., Zhao G., Ling Y., Stoll S.B., Yan Z. (2021). Paper-Based Wearable Electronics. iScience.

[37] Kaur N., Toley B.J. (2018). Paper-Based Nucleic Acid Amplification Tests for Point-of-Care Diagnostics. Analyst.

[38] Sadri B., Goswami D., Sala de Medeiros M., Pal A., Castro B., Kuang S., Martinez R.V. (2018). Wearable and Implantable Epidermal Paper-Based Electronics. ACS Appl. Mater. Interfaces.

[39] Zhang L., Deng H., Yuan R., Yuan Y. (2019). Electrochemical Lead(II) Biosensor by Using an Ion-Dependent Split DNAzyme and a Template-Free DNA Extension Reaction for Signal Amplification. Microchim. Acta.

[40] Wu P., Hwang K., Lan T., Lu Y. (2013). A DNAzyme-Gold Nanoparticle Probe for Uranyl Ion in Living Cells. J. Am. Chem. Soc..

[41] Wang Z., Hao Z., Yu S., Huang C., Pan Y., Zhao X. (2020). A Wearable and Deformable Graphene-Based Affinity Nanosensor for Monitoring of Cytokines in Biofluids. Nanomaterials.

[42] Hao Z., Wang Z., Li Y., Zhu Y., Wang X., de Moraes C.G., Pan Y., Zhao X., Lin Q. (2018). Measurement of Cytokine Biomarkers Using an Aptamer-Based Affinity Graphene Nanosensor on a Flexible Substrate toward Wearable Applications. Nanoscale.

[43] Wang M., Zhang S., Ye Z., Peng D., He L., Yan F., Yang Y., Zhang H., Zhang Z. (2015). A Gold Electrode Modified with Amino-Modified Reduced Graphene Oxide, Ion Specific DNA and DNAzyme for Dual Electrochemical Determination of Pb(II) and Hg(II). Microchim. Acta.

[44] Wang M., Zhai S., Ye Z., He L., Peng D., Feng X., Yang Y., Fang S., Zhang H., Zhang Z. (2015). An Electrochemical Aptasensor Based on a TiO_2_ /Three-Dimensional Reduced Graphene Oxide/PPy Nanocomposite for the Sensitive Detection of Lysozyme. Dalton Trans..

[45] Cai W., Xie S., Zhang J., Tang D., Tang Y. (2017). An Electrochemical Impedance Biosensor for Hg^2+^ Detection Based on DNA Hydrogel by Coupling with DNAzyme-Assisted Target Recycling and Hybridization Chain Reaction. Biosens. Bioelectron..

[46] Gao J., Gao Y., Han Y., Pang J., Wang C., Wang Y., Liu H., Zhang Y., Han L. (2020). Ultrasensitive Label-Free MiRNA Sensing Based on a Flexible Graphene Field-Effect Transistor without Functionalization. ACS Appl. Electron. Mater..

[47] Sheibani S., Capua L., Kamaei S., Akbari S.S.A., Zhang J., Guerin H., Ionescu A.M. (2021). Extended Gate Field-Effect-Transistor for Sensing Cortisol Stress Hormone. Commun. Mater..

[48] Liu Q., Liu Y., Wu F., Cao X., Li Z., Alharbi M., Abbas A.N., Amer M.R., Zhou C. (2018). Highly Sensitive and Wearable In_2_O_3_ Nanoribbon Transistor Biosensors with Integrated On-Chip Gate for Glucose Monitoring in Body Fluids. ACS Nano.

[49] Seshadri D.R., Li R.T., Voos J.E., Rowbottom J.R., Alfes C.M., Zorman C.A., Drummond C.K. (2019). Wearable Sensors for Monitoring the Physiological and Biochemical Profile of the Athlete. NPJ Digit. Med..

[50] Yang B., Fang X., Kong J. (2019). In Situ Sampling and Monitoring Cell-Free DNA of the Epstein-Barr Virus from Dermal Interstitial Fluid Using Wearable Microneedle Patches. ACS Appl. Mater. Interfaces.

[51] Chen B., Li Y., Xu F., Yang X. (2022). Powerful CRISPR-Based Biosensing Techniques and Their Integration With Microfluidic Platforms. Front. Bioeng. Biotechnol..

[52] Bamshad A., Cho H.J. (2021). Laserjet Printed Micro/Nano Sensors and Microfluidic Systems: A Simple and Facile Digital Platform for Inexpensive, Flexible, and Low-Volume Devices. Adv. Mater. Technol..

[53] Ponce Wong R.D., Posner J.D., Santos V.J. (2012). Flexible Microfluidic Normal Force Sensor Skin for Tactile Feedback. Sens. Actuators A Phys..

[54] Park S.-J., Kim J., Chu M., Khine M. (2016). Highly Flexible Wrinkled Carbon Nanotube Thin Film Strain Sensor to Monitor Human Movement. Adv. Mater. Technol..

[55] Song Y., Mukasa D., Zhang H., Gao W. (2021). Self-Powered Wearable Biosensors. Acc. Mater. Res..

[56] Zou Y., Raveendran V., Chen J. (2020). Wearable Triboelectric Nanogenerators for Biomechanical Energy Harvesting. Nano Energy.

[57] Fan W., Shen Z., Zhang Q., Liu F., Fu C., Zhu T., Zhao X. (2022). High-Power-Density Wearable Thermoelectric Generators for Human Body Heat Harvesting. ACS Appl. Mater. Interfaces.

[58] Komkova M.A., Karyakina E.E., Karyakin A.A. (2017). Noiseless Performance of Prussian Blue Based (Bio)Sensors through Power Generation. Anal. Chem..

[59] Zheng H., GhavamiNejad A., GhavamiNejad P., Samarikhalaj M., Giacca A., Poudineh M. (2022). Hydrogel Microneedle-Assisted Assay Integrating Aptamer Probes and Fluorescence Detection for Reagentless Biomarker Quantification. ACS Sens..

[60] Labuda J., Oliveira Brett A.M., Evtugyn G., Fojta M., Mascini M., Ozsoz M., Palchetti I., Paleček E., Wang J. (2010). Electrochemical Nucleic Acid-Based Biosensors: Concepts, Terms, and Methodology (IUPAC Technical Report). Pure Appl. Chem..

[61] Chambers J.P., Arulanandam B.P., Matta L.L., Weis A., Valdes J.J. (2008). Biosensor Recognition Elements. Curr. Issues Mol. Biol..

[62] Morales M.A., Halpern J.M. (2018). Guide to Selecting a Biorecognition Element for Biosensors. Bioconjug. Chem..

[63] Howard G.C., Bethell D.R. (2001). Basic Methods in Antibody Production and Characterization.

[64] Bradbury A.R.M., Trinklein N.D., Thie H., Wilkinson I.C., Tandon A.K., Anderson S., Bladen C.L., Jones B., Aldred S.F., Bestagno M. (2018). When Monoclonal Antibodies Are Not Monospecific: Hybridomas Frequently Express Additional Functional Variable Regions. MAbs.

[65] Kim J., Jeerapan I., Sempionatto J.R., Barfidokht A., Mishra R.K., Campbell A.S., Hubble L.J., Wang J. (2018). Wearable Bioelectronics: Enzyme-Based Body-Worn Electronic Devices. Acc. Chem. Res..

[66] Singh R.S., Singh T., Singh A.K. (2019). Enzymes as Diagnostic Tools. Advances in Enzyme Technology.

[67] Campbell A.S., Kim J., Wang J. (2018). Wearable Electrochemical Alcohol Biosensors. Curr. Opin. Electrochem..

[68] Kvassman J., Pettersson G. (1979). Effect of PH on Coenzyme Binding to Liver Alcohol Dehydrogenase. Eur. J. Biochem..

[69] Sato K., Kang W.H., Saga K., Sato K.T. (1989). Biology of Sweat Glands and Their Disorders. I. Normal Sweat Gland Function. J. Am. Acad. Dermatol..

[70] Wang L., Sun Y. (2021). Engineering Organophosphate Hydrolase for Enhanced Biocatalytic Performance: A Review. Biochem. Eng. J..

[71] Yoo E.-H., Lee S.-Y. (2010). Glucose Biosensors: An Overview of Use in Clinical Practice. Sensors.

[72] Wang F., Li P., Chu H.C., Lo P.K. (2022). Nucleic Acids and Their Analogues for Biomedical Applications. Biosensors.

[73] Du Y., Dong S. (2017). Nucleic Acid Biosensors: Recent Advances and Perspectives. Anal. Chem..

[74] Yang Y., Yang D., Schluesener H.J., Zhang Z. (2007). Advances in SELEX and Application of Aptamers in the Central Nervous System. Biomol. Eng..

[75] Bora U. (2013). Nucleic Acid Based Biosensors for Clinical Applications. Biosens. J..

[76] Zeng R., Wang W., Chen M., Wan Q., Wang C., Knopp D., Tang D. (2021). CRISPR-Cas12a-Driven MXene-PEDOT:PSS Piezoresistive Wireless Biosensor. Nano Energy.

[77] Yu X., Zhang S., Guo W., Li B., Yang Y., Xie B., Li K., Zhang L. (2021). Recent Advances on Functional Nucleic-Acid Biosensors. Sensors.

[78] Chen T.-Y., Loan P.T.K., Hsu C.-L., Lee Y.-H., Tse-Wei Wang J., Wei K.-H., Lin C.-T., Li L.-J. (2013). Label-Free Detection of DNA Hybridization Using Transistors Based on CVD Grown Graphene. Biosens. Bioelectron..

[79] Wang Z., Hao Z., Wang X., Huang C., Lin Q., Zhao X., Pan Y. (2021). A Flexible and Regenerative Aptameric Graphene–Nafion Biosensor for Cytokine Storm Biomarker Monitoring in Undiluted Biofluids toward Wearable Applications. Adv. Funct. Mater..

[80] Ganguly A., Lin K.C., Muthukumar S., Prasad S. (2021). Autonomous, Real-Time Monitoring Electrochemical Aptasensor for Circadian Tracking of Cortisol Hormone in Sub-Microliter Volumes of Passively Eluted Human Sweat. ACS Sens..

[81] Pali M., Jagannath B., Lin K.C., Upasham S., Sankhalab D., Upashama S., Muthukumar S., Prasad S. (2021). CATCH (Cortisol Apta WATCH): ‘Bio-Mimic Alarm’ to Track Anxiety, Stress, Immunity in Human Sweat. Electrochim. Acta.

[82] Mugo S.M., Alberkant J., Bernstein N., Zenkina O.V. (2021). Flexible Electrochemical Aptasensor for Cortisol Detection in Human Sweat. Anal. Methods.

[83] Liu Q., Zhao C., Chen M., Liu Y., Zhao Z., Wu F., Li Z., Weiss P.S., Andrews A.M., Zhou C. (2020). Flexible Multiplexed In_2_O_3_ Nanoribbon Aptamer-Field-Effect Transistors for Biosensing. iScience.

[84] An J.E., Kim K.H., Park S.J., Seo S.E., Kim J., Ha S., Bae J., Kwon O.S. (2022). Wearable Cortisol Aptasensor for Simple and Rapid Real-Time Monitoring. ACS Sens..

[85] Wu Q., Suo C., Brown T., Wang T., Teichmann S.A., Bassett A.R. (2021). INSIGHT: A Population-Scale COVID-19 Testing Strategy Combining Point-of-Care Diagnosis with Centralized High-Throughput Sequencing. Sci. Adv..

[86] Bokelmann L., Nickel O., Maricic T., Pääbo S., Meyer M., Borte S., Riesenberg S. (2021). Point-of-Care Bulk Testing for SARS-CoV-2 by Combining Hybridization Capture with Improved Colorimetric LAMP. Nat. Commun..

[87] Toley B.J., Covelli I., Belousov Y., Ramachandran S., Kline E., Scarr N., Vermeulen N., Mahoney W., Lutz B.R., Yager P. (2015). Isothermal Strand Displacement Amplification (ISDA): A Rapid and Sensitive Method of Nucleic Acid Amplification for Point-of-Care Diagnosis. Analyst.

[88] Ciftci S., Neumann F., Abdurahman S., Appelberg K.S., Mirazimi A., Nilsson M., Madaboosi N. (2020). Digital Rolling Circle Amplification–Based Detection of Ebola and Other Tropical Viruses. J. Mol. Diagn..

[89] Oliveira B.B., Veigas B., Baptista P.V. (2021). Isothermal Amplification of Nucleic Acids: The Race for the Next “Gold Standard”. Front. Sens..

[90] Müller S., Strohbach D., Wolf J. (2006). Sensors Made of RNA: Tailored Ribozymes for Detection of Small Organic Molecules, Metals, Nucleic Acids and Proteins. IEE Proc. Nanobiotechnol..

[91] Yao C., Zhu T., Qi Y., Zhao Y., Xia H., Fu W. (2010). Development of a Quartz Crystal Microbalance Biosensor with Aptamers as Bio-Recognition Element. Sensors.

[92] Chen Y., Zhu Q., Zhou X., Wang R., Yang Z. (2021). Reusable, Facile, and Rapid Aptasensor Capable of Online Determination of Trace Mercury. Environ. Int..

[93] Ly T.T., Ruan Y., Du B., Jia P., Zhang H. (2021). Fibre-Optic Surface Plasmon Resonance Biosensor for Monoclonal Antibody Titer Quantification. Biosensors.

[94] Lee J.-O., So H.-M., Jeon E.-K., Chang H., Won K., Kim Y.H. (2008). Aptamers as Molecular Recognition Elements for Electrical Nanobiosensors. Anal. Bioanal. Chem..

[95] Yu M., Chang Q., Zhang L., Huang Z., Song C., Chen Y., Wu X., Lu Y. (2022). Ultra-sensitive Detecting OPs-Isocarbophos Using Photoinduced Regeneration of Aptamer-based Electrochemical Sensors. Electroanalysis.

[96] Knott G.J., Doudna J.A. (2018). CRISPR-Cas Guides the Future of Genetic Engineering. Science.

[97] Gootenberg J.S., Abudayyeh O.O., Lee J.W., Essletzbichler P., Dy A.J., Joung J., Verdine V., Donghia N., Daringer N.M., Freije C.A. (2017). Nucleic Acid Detection with CRISPR-Cas13a/C2c2. Science.

[98] Kaminski M.M., Abudayyeh O.O., Gootenberg J.S., Zhang F., Collins J.J. (2021). CRISPR-Based Diagnostics. Nat. Biomed. Eng..

[99] Al Mamun M., Wahab Y.A., Hossain M.A.M., Hashem A., Johan M.R. (2021). Electrochemical Biosensors with Aptamer Recognition Layer for the Diagnosis of Pathogenic Bacteria: Barriers to Commercialization and Remediation. TrAC Trends Anal. Chem..

[100] Chen C., Wang J. (2020). Optical Biosensors: An Exhaustive and Comprehensive Review. Analyst.

[101] Damborský P., Švitel J., Katrlík J. (2016). Optical Biosensors. Essays Biochem..

[102] Sedki M., Shen Y., Mulchandani A. (2020). Nano-FET-Enabled Biosensors: Materials Perspective and Recent Advances in North America. Biosens. Bioelectron..

[103] Wu Y., Ghoraani B. (2022). Biological Signal Processing and Analysis for Healthcare Monitoring. Sensors.

[104] Zhang A., Lieber C.M. (2016). Nano-Bioelectronics. Chem. Rev..

[105] Vu C.A., Chen W.Y. (2019). Field-Effect Transistor Biosensors for Biomedical Applications: Recent Advances and Future Prospects. Sensors.

[106] Sadighbayan D., Hasanzadeh M., Ghafar-Zadeh E. (2020). Biosensing Based on Field-Effect Transistors (FET): Recent Progress and Challenges. TrAC-Trends Anal. Chem..

[107] Nakatsuka N., Yang K.-A., Abendroth J.M., Cheung K.M., Xu X., Yang H., Zhao C., Zhu B., Rim Y.S., Yang Y. (2018). Aptamer-Field-Effect Transistors Overcome Debye Length Limitations for Small-Molecule Sensing. Science.

[108] Stern E., Wagner R., Sigworth F.J., Breaker R., Fahmy T.M., Reed M.A. (2007). Importance of the Debye Screening Length on Nanowire Field Effect Transistor Sensors. Nano Lett..

[109] Zea M., Bellagambi F.G., ben Halima H., Zine N., Jaffrezic-Renault N., Villa R., Gabriel G., Errachid A. (2020). Electrochemical Sensors for Cortisol Detections: Almost There. TrAC Trends Anal. Chem..

[110] Menon S., Mathew M.R., Sam S., Keerthi K., Kumar K.G. (2020). Recent Advances and Challenges in Electrochemical Biosensors for Emerging and Re-Emerging Infectious Diseases. J. Electroanal. Chem..

[111] Rafat N., Satoh P., Worden R.M. (2021). Electrochemical Biosensor for Markers of Neurological Esterase Inhibition. Biosensors.

[112] Yang B., Fang X., Kong J. (2020). Engineered Microneedles for Interstitial Fluid Cell-Free DNA Capture and Sensing Using Iontophoretic Dual-Extraction Wearable Patch. Adv. Funct. Mater..

[113] Kim J., Sempionatto J.R., Imani S., Hartel M.C., Barfidokht A., Tang G., Campbell A.S., Mercier P.P., Wang J. (2018). Simultaneous Monitoring of Sweat and Interstitial Fluid Using a Single Wearable Biosensor Platform. Adv. Sci..

[114] Bariya M., Nyein H.Y.Y., Javey A. (2018). Wearable Sweat Sensors. Nat. Electron..

[115] Manjakkal L., Yin L., Nathan A., Wang J., Dahiya R. (2021). Energy Autonomous Sweat-Based Wearable Systems. Adv. Mater..

[116] Anwer A.H., Khan N., Ansari M.Z., Baek S.-S., Yi H., Kim S., Noh S.M., Jeong C. (2022). Recent Advances in Touch Sensors for Flexible Wearable Devices. Sensors.

[117] Sekar M., Sriramprabha R., Sekhar P.K., Bhansali S., Ponpandian N., Pandiaraj M., Viswanathan C. (2020). Review—Towards Wearable Sensor Platforms for the Electrochemical Detection of Cortisol. J. Electrochem. Soc..

[118] Shaver A., Arroyo-Currás N. (2022). The Challenge of Long-Term Stability for Nucleic Acid-Based Electrochemical Sensors. Curr. Opin. Electrochem..

[119] Singh N.K., Chung S., Sveiven M., Hall D.A. (2021). Cortisol Detection in Undiluted Human Serum Using a Sensitive Electrochemical Structure-Switching Aptamer over an Antifouling Nanocomposite Layer. ACS Omega.

